# Complete Genome Sequence of the Marine Hydrocarbon Degrader Alcaligenes aquatilis QD168, Isolated from Crude Oil-Polluted Sediment of Quintero Bay, Central Chile

**DOI:** 10.1128/MRA.01664-18

**Published:** 2019-01-31

**Authors:** Roberto E. Durán, Bárbara Barra-Sanhueza, Francisco Salvà-Serra, Valentina Méndez, Daniel Jaén-Luchoro, Edward R. B. Moore, Michael Seeger

**Affiliations:** a Molecular Microbiology and Environmental Biotechnology Laboratory, Department of Chemistry & Centro de Biotecnología Daniel Alkalay Lowitt, Universidad Técnica Federico Santa María, Valparaíso, Chile; b Department of Infectious Diseases, Institute for Biomedicine, Sahlgrenska Academy, University of Gothenburg, Gothenburg, Sweden; c Culture Collection University of Gothenburg (CCUG), Sahlgrenska University Hospital and Sahlgrenska Academy, University of Gothenburg, Gothenburg, Sweden; d Centre for Antibiotic Resistance Research (CARe), University of Gothenburg, Gothenburg, Sweden; e Microbiology, Department of Biology, University of the Balearic Islands, Palma de Mallorca, Spain; Loyola University Chicago

## Abstract

Alcaligenes aquatilis strain QD168 (= CCUG 69566) is a marine hydrocarbon-degrading bacterium isolated from crude oil-polluted sediment from Quintero Bay, Central Chile. Here, we present the 4.32-Mb complete genome sequence of strain QD168, with 3,892 coding sequences, 58 tRNAs, and a 56.3% G+C content.

## ANNOUNCEMENT

Members of the genus Alcaligenes are Gram-negative, aerobic, and motile bacteria that are frequently isolated from water, soil, and clinical samples, as well as from industrial and contaminated environments (1–6). Alcaligenes strains have been associated with the degradation of toxic aromatic compounds and xenobiotics, including phenol (1), atrazine (2), endosulfan (3), phenanthrene (4), pyrene, chrysene, and benzo[*a*]pyrene (5). Here, we report the complete genome sequence of Alcaligenes aquatilis QD168 (= CCUG 69566), a marine hydrocarbon-degrading bacterium capable of growing on *n*-hexadecane and diesel in M9 minimal medium agar (7) at 30°C. A. aquatilis strain QD168 was isolated from a crude oil-polluted marine sediment sample from Quintero Bay, Central Chile by enrichment in diesel (1% vol/vol) in Bushnell Hass mineral medium and artificial seawater.

Genomic DNA of QD168 cells cultivated in Luria-Bertani broth was extracted using a Qiagen Genomic-tip 100/G kit (Qiagen, Germany). Next-generation sequencing was performed using a PacBio RS II platform (Uppsala Genome Center, Sweden) with one single-molecule real-time (SMRT) cell and an average 20-kb insert library, obtaining 87,836 reads with an average length of 12,761 bp. PacBio reads were trimmed and assembled using the Hierarchical Genome Annotation Pipeline (HGAP) v3.0 (8), obtaining an initial assembly of 3 contigs. The contigs were checked for overlapping regions by dot plot analysis using Gepard v1.40 (9), obtaining one contig. The genome of strain QD168 consists of a 4,323,879-bp circular chromosome (168× coverage) with a 56.3% G+C content.

Gene functional annotation was performed using the NCBI Prokaryotic Genome Annotation Pipeline (PGAP) v4.6 (10), identifying 3,892 coding sequences (CDS), 3 rRNA operons, 58 tRNAs, 4 noncoding RNAs (ncRNAs), and 2 CRISPR arrays. QD168 genome analysis with the Comprehensive Antibiotic Resistance Database (CARD) v3.0.0 (11) identified one antibiotic resistance gene (locus tag D3M96_11220), possessing 43% identity with a resistance-nodulation-cell division (RND) antibiotic efflux pump for fluoroquinolone and tetracycline. Genes were identified by BLAST using the UniProt-KB/Swiss-Prot database (>30% identity). Biosynthetic genes for the osmoprotectants ectoine and 5-hydroxyectoine (*ectABCD*; D3M96_13270, D3M96_13265, D3M96_13260, and D3M96_13255) and the bioplastic polyhydroxyalkanoates (*phaBC*; D3M96_06600 and D3M96_06595) were identified. Aromatic catabolic genes encoding catechol 1,2-dioxygenase (*catA*; D3M96_09460), catechol 2,3-dioxygenase (*catE*; D3M96_10490), homogentisate 1,2-dioxygenase (*hmgA*; D3M96_03780), protocatechuate 4,5-dioxygenase (*ligAB*; D3M96_07060, D3M96_07055) and gentisate 1,2-dioxygenase (*sdgD*; D3M96_00260) were identified.

Comparative 16S rRNA gene sequence analyses using EMBOSS Needle (https://www.ebi.ac.uk/Tools/psa/emboss_needle/) showed a close relationship (99.9% identity) with A. aquatilis LMG 22996^T^, while identities with those of the type strains of other Alcaligenes species were <98.5% (species threshold) (12). Multilocus sequence analysis (MLSA) was performed with the type strains of four Alcaligenes species. 16S rRNA gene, *gyrB*, and *nirK* sequences were aligned by Clustal W v.1.81 (13) and manually curated and concatenated, obtaining similarities of 97.5% to A. aquatilis LMG 22996^T^ and 94.5% to A. faecalis DSM 30030^T^. Genome sequence analysis by average nucleotide identity, based on BLAST (ANIb) (14), using JSpeciesWS v.3.0.2 (15) with A. aquatilis BU33N (GenBank accession number GCA_003076515; identical 16S rRNA gene sequence with that of A. aquatilis LMG 22996^T^) and A. faecalis DSM 30030^T^, resulted in ANIb values of 96.8% and 90.5% (species threshold, >95 to 96%), respectively.

This is the first complete genome sequence of an A. aquatilis strain.

### Data availability.

The genome sequence of Alcaligenes aquatilis strain QD168 has been deposited in DDBJ/ENA/GenBank under the accession number CP032153. The version described in this paper is the first version, CP032153.1. The accession number for the publicly available raw data at NCBI is PRJNA489687.
